# Indoxyl Sulfate Induces Renal Fibroblast Activation through a Targetable Heat Shock Protein 90-Dependent Pathway

**DOI:** 10.1155/2019/2050183

**Published:** 2019-04-17

**Authors:** Samantha Milanesi, Silvano Garibaldi, Michela Saio, Giorgio Ghigliotti, Daniela Picciotto, Pietro Ameri, Giacomo Garibotto, Chiara Barisione, Daniela Verzola

**Affiliations:** ^1^University of Genova and IRCCS Ospedale Policlinico San Martino Nephrology, Dialysis and Transplantation Division, Genova, Italy; ^2^Laboratory of Cardiovascular Biology, IRCCS Ospedale Policlinico San Martino & Department of Internal Medicine, University of Genova, Genova, Italy; ^3^Center of Excellence for Biomedical Research, University of Genova, Genova, Italy

## Abstract

Indoxyl sulfate (IS) accumulation occurs early during chronic kidney disease (CKD) progression and contributes to renal dysfunction by inducing fibrosis, inflammation, oxidative stress, and tissue remodeling. Renal toxicity of high IS concentrations (250 *μ*M) has been widely explored, particularly in resident tubular and glomerular cells, while the effect of a moderate IS increase on kidneys is still mostly unknown. To define the effects of IS accumulation on renal fibroblasts, we first analyzed kidneys of C57BL/6 mice receiving IS (0.1%) in drinking water for 12 weeks. As a next step, we treated renal fibroblasts (NRK-49F) with IS (20 *μ*M) with or without the HSP90 inhibitor 17-AAG (1 *μ*M). In mouse kidneys, IS increased the collagen deposition and HSP90 and *α*-SMA expression (immunohistochemistry) in interstitial fibroblasts and caused tubular necrosis (histological H&E and picrosirius red staining). In NRK-49F cells, IS induced MCP1, TGF-*β*, collagen I, *α*-SMA, and HSP90 gene/protein expression and Smad2/3 pathway activation. IS had no effects on fibroblast proliferation and ROS production. 17-AAG counteracted IS-induced MCP1, TGF-*β*, collagen I, and *α*-SMA expression and Smad2/3 phosphorylation. Our study demonstrates that the IS increase promotes renal fibroblast activation by a HSP90-dependent pathway and indicates HSP90 inhibition as a potential strategy to restrain IS-induced kidney inflammation and fibrosis in CKD.

## 1. Introduction

In patients with chronic kidney disease (CKD), the progressive decline of the glomerular filtration rate (GFR) and kidney metabolic function hinders the removal of several endogenous toxins which are normally cleared by the kidney. A current hypothesis is that these toxic compounds, accumulating in blood and tissues, become triggers for CKD progression and contribute to CKD-related complications.

Indoxyl sulfate (IS) has been extensively studied as a putative uremic toxin [1, 2]. Circulating IS increases rather precociously in CKD patients [3] and reaches very high plasma levels in patients with stage 5-5D CKD, exceeding 500 *μ*M/l as compared to 0.1–2.39 *μ*M/L in the healthy population [4]. Remarkably, previous in vitro observations reveal that even a moderate increase in IS affects cell homeostasis and induces tissue remodeling [5], and several clinical studies point out that IS levels predict the progression of CKD [6].

Renal toxicity of high IS concentrations (exceeding 250 *μ*M) has been widely explored, in particular in resident tubular and glomerular cells. Both in proximal tubule cells and in podocytes [7], IS has profibrotic [8], prooxidant [9], and proinflammatory [10] action, while the effect of a moderate increase in circulating IS levels in kidneys is far to be defined. Renal fibrosis is a common adaptive response to a variety of pathological triggers, and fibroblast activation in the kidney contributes to tissue remodeling by collagen production and release of profibrotic factors [11], being involved in the activation of multiple pathways which include the TGF-*β* and the Smad downward signaling [12].

Heat shock proteins (HSPs) are a family of molecular chaperone proteins; among them, HSP90 is one of the most abundant and is involved in protein folding and stabilization [13]. Various stressful conditions induce the activation of HSP90, which has been found to be upregulated during the ischemia-reperfusion injury in the kidney [14] and in models of dermal [15] and pulmonary fibrosis [16]. IS is a ligand of the aryl hydrocarbon receptor (AhR); upon binding, IS and AhR form a complex with HSP90, which translocates to the nucleus and promotes proinflammatory and fibrotic target gene transcription.

In this paper, we initially observed the effect of IS supplementation on kidney histology, HSP90 expression, and fibroblast phenotype in mice. Next, we characterized *in vitro* the effects of 20 *μ*M IS, a concentration found in early stages of CKD, on the renal fibroblast phenotype and inflammatory profile through activation of Smad 2/3 and HSP90. Finally, we demonstrated that pretreatment with 17-N-allylamino-17demethoxygeldanamycin (17-AAG), a selective HSP90 inhibitor, is able to reverse the IS-induced fibroblast activation, suggesting HSP90 inhibition as an option to restrain fibrosis in kidneys during CKD progression.

## 2. Materials and Methods

### 2.1. Animal Model

**Figure 1:**
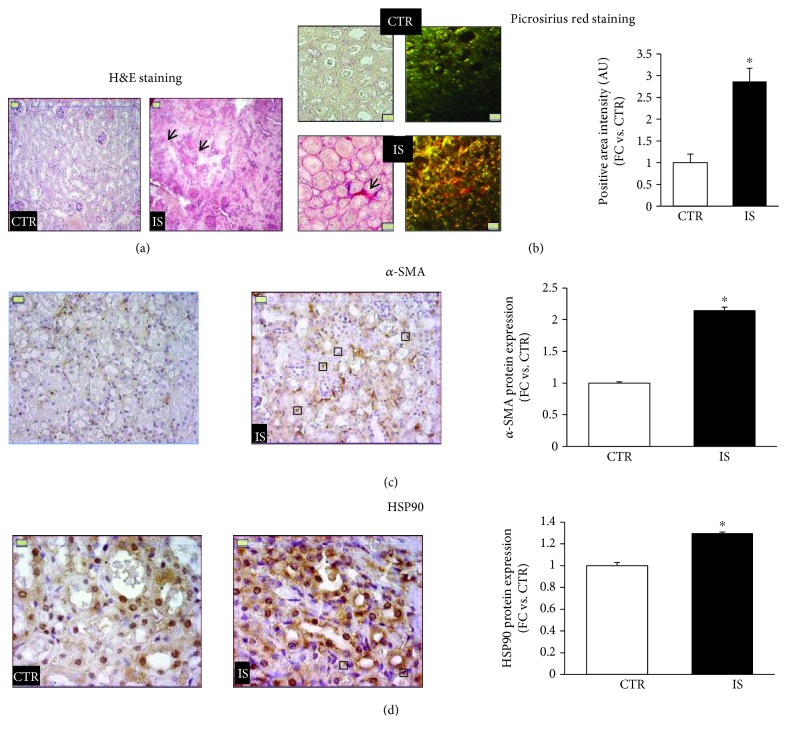
Mouse kidney analysis after a 12-week exposure to 0.1% IS. (a) H&E staining; arrows indicate areas of tubular necrosis. (b) Collagen deposition as revealed by picrosirius red staining, brightfield illumination (left side) and polarization contrast illumination (right side); the graph represents the quantification of red staining intensity under brightfield illumination. (c, d) Images of immunochemistry of *α*-SMA and HSP90; boxes specify the positive nuclei of interstitial fibroblasts found in the kidney from IS-treated mice. The histograms indicate the immunopositivity quantification (fold change: FC). (magnification: (a)–(c), ×400; (d), ×1000); ^∗^ *p* < 0.05.

Male C57BL/6 mice (bred in-house from stock originally obtained from Harlan Laboratories, S. Pietro al Natisone, UD, Italy), 8 to 12 weeks old, were housed in a pathogen-free environment. Water and regular mouse diet were available ad libitum. Two separate groups were used to acquire data: the IS group (IS, *N* = 6), receiving 0.1% IS in drinking water, and the control group (CTR, *N* = 6). Water consumption was recorded every second day, when replacing the IS solution, and the body weight weekly. The study lasted for 12 weeks (Supplementary 1, Figure 1A). At the termination of the in vivo study, mice were killed in a CO_2_ chamber. Immediately after explantation, kidneys were dissected and kept in cold PBS for 40 minutes, changing the buffer solution 3 times, and then fixed in cold 2% paraformaldehyde.

### 2.2. Histopathological Examination and Fibrosis Quantification

Standard histopathological techniques were followed for processing the fixed kidney tissue and the preparation of paraffin blocks. Hematoxylin and eosin (H&E) staining was performed to detect tissue damage and tubular necrosis. Specimens were examined in a blinded manner by two pathologists independently under light microscopy. Briefly, six high-power fields (40x magnification) were checked for confluent cell necrosis or sloughing of the tubular epithelium and loss of nuclei and of cytoplasm (evidenced as light areas), as described by Speir and colleagues [17].

Picrosirius red staining was performed to quantify collagen deposition. The paraffin sections were dewaxed, hydrated, and stained with a picrosirius red solution (0.1% sirius red F3B in saturated picric acid) for 1 h, washed twice in acidified water, and counterstained with Carazzi's hematoxylin.

### 2.3. Immunohistochemistry

Paraffin sections (5 *μ*m) of 2% paraformaldehyde-fixed tissue were analyzed for an HSP90 and nitrotyrosine mouse monoclonal antibody (Santa Cruz Biotechnology, Dallas, Texas, USA) and *α*-SMA mouse monoclonal antibody (Dako Agilent Pathology Solution, Santa Clara, USA). Immunostaining was performed as described previously [18].

### 2.4. Cell Cultures and Treatments

Rat kidney fibroblast cells (NRK-49F) were obtained from the American Type Culture Collection (ATCC, Carlsbad, California, USA). NRK-49F cells were maintained in DMEM (EuroClone, Milan, Italy) containing 2 mmol L-glutamine and 100 U/mL penicillin-streptomycin (EuroClone), with 5% FBS, and incubated at 37°C with 5% of CO_2_.

NRK-49F cells were incubated with 20 *μ*M IS, diluted in ultrapure H_2_O, for different time lags, depending on the experimental requirements: the cells were incubated for 15, 30, and 120 minutes to assess the activation of HSP90 and p- (phospho-) Smad 2/3, for 5 hours to evaluate the mRNA levels of MCP1, TGF-*β*, and collagen I, for 1, 3, and 16 hours to evaluate the mRNA levels of NOX4, and for 24 hours to evaluate *α*-SMA and collagen I protein expression. Finally, fibroblasts were treated with 20 *μ*M IS for 60 minutes to assess the ROS production and for 24 and 48 hours to analyze the cell proliferation. In selected experiments, 1 *μ*M HSP90 inhibitor (tanespimycin (17-AAG); Selleckchem, Munich, Germany) was added to the culture medium 1 hour before stimulation with IS.

### 2.5. mRNA Analysis

The total RNA was extracted using the QIAzol Lysis Reagent (Qiagen Sciences, Maryland, USA), and the concentration and integrity of each sample were evaluated on a NanoDrop ND-1000 Spectrophotometer (NanoDrop Technologies Inc., Wilmington, DE, USA). 1 *μ*g RNA was used for cDNA synthesis.

### 2.6. cDNA Reverse Transcription and Quantitative Real-Time PCR

cDNA synthesis was performed using the iScript™ cDNA synthesis kit RT (Bio-Rad Laboratories Inc., Hercules, California, USA). MCP1, TGF-*β*, collagen I, NOX4, and GAPDH primers were obtained from Tib Molbiol Srl (Genoa, Italy), and sequences are reported in Table 1. PCR amplification was carried out in a total volume of 10 *μ*l, containing 1 *μ*l of cDNA solution, 5 *μ*l of SYBR Master Mix solution (Eppendorf, Hamburg, Germany), 0.03 *μ*l of each primer, and 3.94 *μ*l of nuclease-free water. GAPDH was quantified and used for the normalization of expression values of the other genes. Fluorescence signals measured during the amplification were considered positive if the fluorescence intensity was more than 20-fold greater than the standard deviation of the baseline fluorescence. The ∆∆CT method of relative quantification was used to determine the fold change in expression. Assays were run in triplicate using Universal PCR Master Mix on a MasterCycler RealPlex (Eppendorf) PCR system.

### 2.7. Proliferation and ROS Production

Proliferation was evaluated by cell labeling with carboxyfluorescein succinimidyl ester (CFDA-SE; Invitrogen, Carlsbad, California, USA). Data were analyzed with the Proliferation Wizard module of the ModFit LT 4.0 software (Verity Software House, Topsham, ME, USA), and the results were expressed as the proliferation index.

Intracellular ROS production was evaluated using the CellROX Deep Red kit from Life Technologies (Carlsbad, California, USA). Following treatments, a CellROX reagent was added for 30 minutes. Cells were directly analyzed on FACSCanto II.

### 2.8. Western Blot Analysis

Cells were lysed in cold buffer (20 mM HEPES, 150 mM NaCl, 10% (*v*/*v*) glycerol, 0.5% (*v*/*v*) NP-40, 1 mM EDTA, 2.5 mM DTT, 10 *μ*g/l aprotinin, leupeptin, pepstatin A, 1 mM PMSF, and Na3VO4). Protein concentration was determined by using the Pierce BCA Protein Assay Kit (Thermo Fisher Scientific; Rockford, IL, USA), and 10–20 *μ*g was resolved on SDS-polyacrylamide gels and electrotransferred to a PVDF membrane (Merck Group). Blots were probed using an anti-HSP90 (AC88) monoclonal antibody (Santa Cruz Biotechnology), anti-p-SMAD 2 (Ser 465/467) polyclonal antibody (Cell Signaling Technology; Danvers, USA), anti-p-SMAD 3 (Ser 423/425) monoclonal antibody (Cell Signaling Technology), anti-*α*-SMA monoclonal antibody (Dako Agilent Pathology Solution; Santa Clara, CA, USA), anti-TGF-*β* monoclonal antibody (Santa Cruz Biotechnology), and anti-CCL2/MCP1 polyclonal antibody (Novus Biologicals). The reference proteins were detected with an anti-*β*-actin mouse monoclonal antibody (Santa Cruz Biotechnology), anti-Smad rabbit polyclonal antibody (Cell Signaling Technology), or anti-histone 3 rabbit polyclonal antibody (Cell Signaling Technology) and incubated with horseradish peroxidase secondary antibodies (Cell Signaling Technology). Immunoblots were developed with the Immobilon Western Chemiluminescent HRP Substrate (Merck Group, Darmstadt, Germany). Band intensities were determined using the Alliance system (Uvitec; Cambridge, UK).

### 2.9. Immunocytochemistry and Immunofluorescence

NRK-49F cells were grown on chamber slides to subconfluence, treated with IS with or without 17-AAG and fixed in cold methanol. For immunocytochemistry, after a 24-hour treatment, fixed cells had undergone quenching of the endogenous peroxidase with H_2_O_2_ in PBS and incubation with an anti-collagen I polyclonal antibody (Proteintech; Manchester, UK) and with an anti-*α*-SMA monoclonal antibody (Dako Agilent Pathology Solution); immunostaining was then performed as previously described [19]. For immunofluorescence, treatments lasted for 15, 45, and 120 min. Cells were incubated with an anti-HSP90 monoclonal antibody (AC88, Santa Cruz Biotechnology) and then with the secondary anti-mouse antibody goat-anti-mouse Alexa Fluor 488 (Invitrogen, Carlsbad, CA, USA).

### 2.10. Image Analysis

In immunohistochemical/immunocytochemical staining, the positivity was evaluated by image analysis performed using the Leica Q500 MC Image Analysis System (Leica, Cambridge, UK) as previously described [18]. For picrosirius red staining, a total of 10 fields were randomly chosen per mouse and images were viewed with brightfield illumination as well as with polarization contrast illumination at 40x. Picrosirius red is a birefringent molecule that binds to collagens. The complex fibrillar collagen/sirius red can be detected under polarized light, and the collagen bundles are red, yellow, and green. Collagen expression was quantified under brightfield illumination.

### 2.11. Statistics

In vitro experiments were performed at least 3 times. Summary data are expressed as mean ± SEM and compared by Student's *t*-test. Statistical significance was set at *p* < 0.05. All statistical analyses were performed using GraphPad Prism version 5.00 for Windows (GraphPad Software, San Diego, California, USA).

## 3. Results

### 3.1. IS Induces Fibrosis and HSP90 Activation in Mice

Kidneys of C57BL/6 mice receiving continuous supplementation of 0.1% IS in drinking water displayed signs of tubular necrosis (Figure 1(a)) and interstitial fibrosis whose severity was evaluated by picrosirius red staining (Figure 1(b)). As shown in Figure 1(b), a significant increase in staining intensity was noticeable in the interstitial zone of kidneys obtained from IS-treated mice (3 fold in respect top untreated control mice, *p* < 0.01). *α*-SMA expression in the interstitium is associated with the progression of kidney disease, and the accumulation of *α*-SMA-positive fibroblasts represents the earliest histological marker of fibrosis progression [20]. We found a significant increase in *α*-SMA protein in interstitial fibroblasts (2-fold vs. control mice, *p* < 0.05) (Figure 1(c)) and upregulation of HSP90 in the tubular-interstitial compartment (1.4-fold increase vs. control mice, *p* < 0.05) (Figure 1(d)). These data revealed that even moderate IS increases may induce HSP90 overexpression and prime interstitial fibroblasts to an activated phenotype.

### 3.2. IS Induces a Profibrotic and Proinflammatory Phenotype in NRK49F Cells

Next, we examined the effects of 20 *μ*M IS on the profibrotic and proinflammatory phenotype in the NRK-49F cell line.

**Figure 2:**
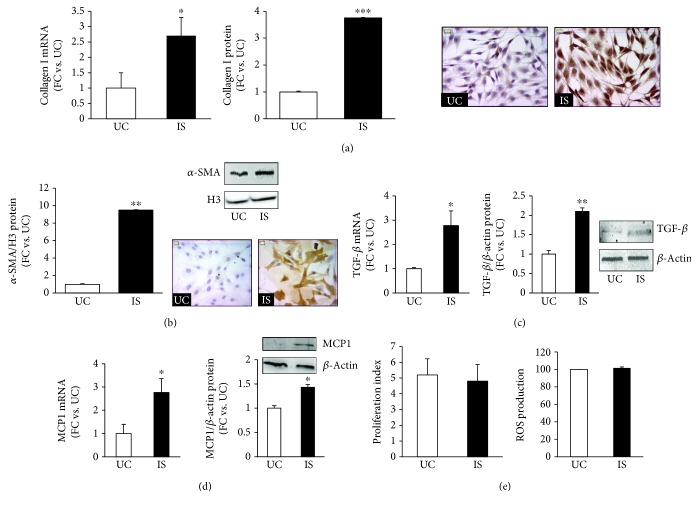
Effects of IS on the NRK-49F rat kidney fibroblast cell line. (a) Collagen I mRNA and protein in IS-treated and untreated NRK-49F cells. Data are expressed as fold change (FC) ± SEM versus untreated cells (^∗^ *p* < 0.05 and ^∗∗∗^ *p* < 0.001, respectively). Photos are representative of collagen I expression and pattern evaluated by immunocytochemistry (magnification ×400). (b) *α*-SMA protein expression in IS-treated and untreated NRK-49F cells. Data, obtained by western blot analysis, are expressed as fold change (FC) ± SEM versus untreated cells (^∗∗^ *p* < 0.01). Pictures are representative of *α*-SMA expression evaluated by western blot and immunocytochemistry. (c) TGF-*β* and (d) MCP1 mRNA and protein levels in IS-treated or untreated NRK-49F cells. mRNA expression is evaluated by real-time PCR, normalized for GAPDH mRNA. Western blot analysis is normalized for *β*-actin; values are expressed as fold increase ± SEM versus untreated cells (^∗^ *p* < 0.05). (e) Flow cytometry analysis of the proliferation index, after 48 hours of exposure, and ROS production, after 60 minutes of exposure, as detected by the probes CFSE and CellRox, respectively, in NRK-49F cells treated or not with 20 *μ*M IS. UC: untreated cells; IS: indoxyl sulfate-treated cells.

Both collagen I mRNA and protein were increased 3-fold (*p* < 0.05 and 0.001, respectively) (Figure 2(a)), and *α*-SMA protein level arose significantly, as shown by western blot analysis and immunocytochemistry (Figure 2(b): 9.5-fold, *p* < 0.01), in respect to untreated control cells.

IS also induced expression of TGF-*β* (Figure 2(c): 2.8-fold for mRNA and 2-fold for protein expression vs. untreated cells; *p* < 0.05 and 0.01, respectively), a potent mediator in renal fibrosis, and MCP1, one of the inflammatory cytokines involved in tubular-interstitial injury (3-fold for mRNA and 1.5-fold for protein expression vs. untreated cells, *p* < 0.05) (Figure 2(d)).

### 3.3. IS Does Not Stimulate Proliferation and Oxidative Stress in NRK49 Cells

Proliferation of fibroblasts and oxidative stress are critical pathological processes during initiation and maintenance of fibrotic lesions; however, in our experimental setting, 20 *μ*M IS did not significantly affect the proliferation rate neither the redox status as revealed by ROS production (Figure 2(e)) and NOX4 mRNA (Supplementary 2, Figure 2).

### 3.4. Effects of IS on Cell Signaling

**Figure 3:**
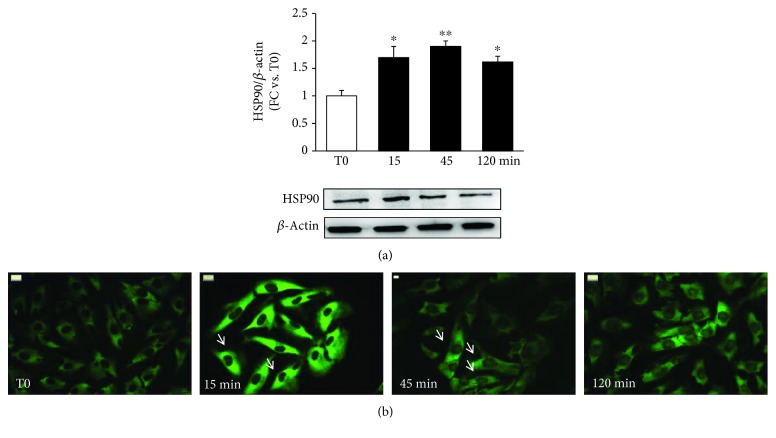
Effects of IS on HSP90 after different times of IS exposure (0, 15, 45, and 120 min). (a) Total protein expression, evaluated by western blot, and (b) intracellular localization, detected by immunofluorescence. Arrows indicate perinuclear localization (^∗^ *p* < 0.05, ^∗∗^ *p* < 0.01).

**Figure 4:**
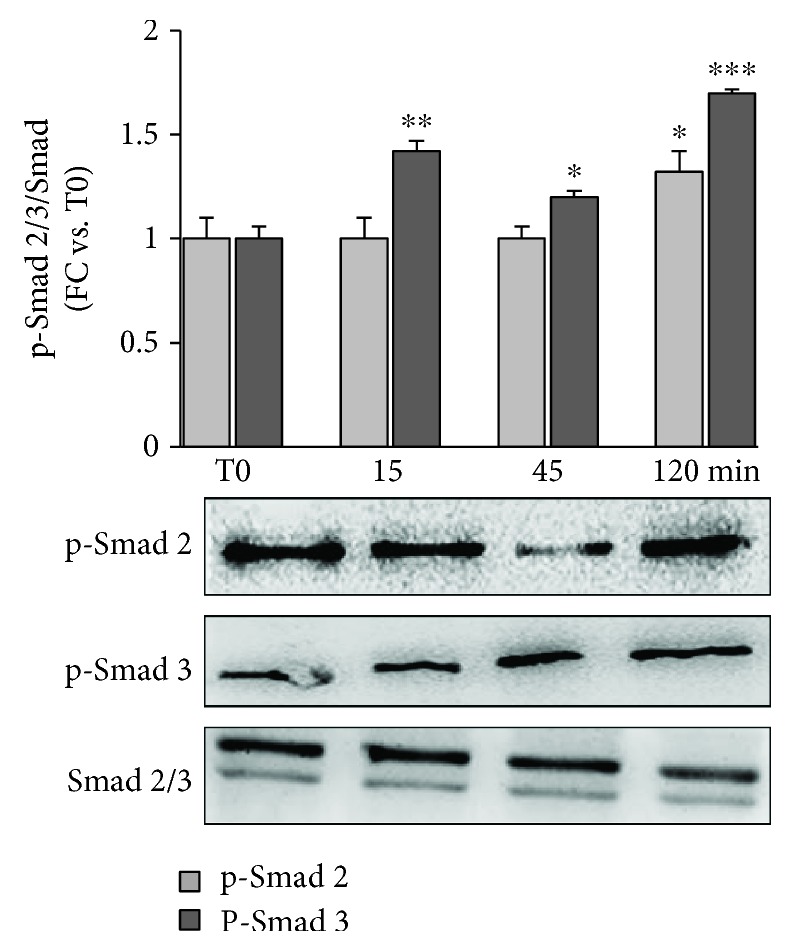
Smad 2 and 3 phosphorylation after different times of IS exposure (15, 45, and 120 min) in respect to untreated cells (T0), normalized for total Smad 2/3. Measures are represented as fold changes ± SEM versus untreated cells (^∗^ *p* < 0.05, ^∗∗^ *p* < 0.01, and ^∗∗∗^ *p* < 0.001).

To confirm our observation on mouse kidneys, we verified in NRK-49F cells the effects of IS on HSP90 activation. IS induced a 70% increase in HSP90 protein expression after a 15-minute treatment; this increase in respect to the control level lasted 2 hours (*p* < 0.01-0.05 vs. baseline referred to as T0) (Figure 3(a)). By looking at the intracellular distribution, a marked perinuclear concentration of HSP90 was noticeable already after a 15-minute IS treatment (Figure 3(b)); looking at the downstream signaling, we examined the effects of IS on the Smad pathway (Figure 4). Both Smad 2 and Smad 3 are activated in respect to T0 after 120 minutes (*p* < 0.05 and *p* < 0.001, respectively) with Smad 3 phosphorylation occurring early within 15 minutes (*p* < 0.01).

### 3.5. Effects of HSP90 Inhibition on the Profibrotic and Proinflammatory Phenotype

**Figure 5:**
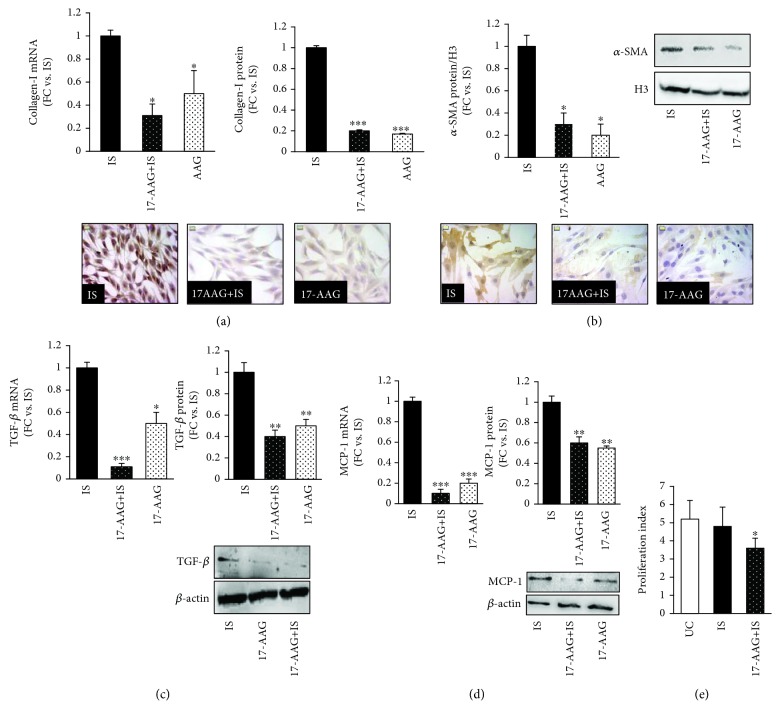
Effects of HSP90 inhibition on the profibrotic and proinflammatory phenotype of NRK-49F cells. (a) Collagen I mRNA and protein levels and representative images (magnification 400x). The protein expression is evaluated by immunocytochemistry, as shown in the pictures. Values are expressed as fold changes ± SEM versus cells treated with IS. (b) Western blot and immunocytochemistry for *α*-SMA protein expression. The graph reports the measure obtained from western blot analysis. (c) TGF-*β* and (d) MCP1 mRNA and protein expression. (e) Proliferation index of IS and 17-AAG+IS cells vs. untreated cells (^∗^ *p* < 0.05, ^∗∗∗^ *p* < 0.001). mRNA expression is tested by real-time PCR and normalized to GAPDH mRNA. UC: untreated cells; IS: indoxyl sulfate-treated cells; 17-AAG+IS: cells pretreated with the HSP90 inhibitor 17-AAG and then treated with IS.

We next examined the effects of IS treatment in the presence of 1 *μ*M 17-AAG, a selective inhibitor of HSP90. 17-AAG counteracted the increase in mRNA and protein collagen I synthesis (-70 and -80%, respectively, *p* < 0.05-0.001) (Figure 5(a)) and in *α*-SMA protein expression (*p* < 0.05) as depicted in Figure 5(b). The inhibition of HSP90 also blunted TGF-*β* and MCP1 mRNA (-90%, *p* < 0.001) and protein expression (-60% and -40%, respectively, *p* < 0.01) (Figures 5(c) and 5(d)). Lastly, 17-AAG lowered NRK-49F cells proliferation (*p* < 0.05 vs. IS-treated cells) (Figure 5(e)).

### 3.6. 17-AAG Blocks IS-Induced Activation of the Smad Pathway

**Figure 6:**
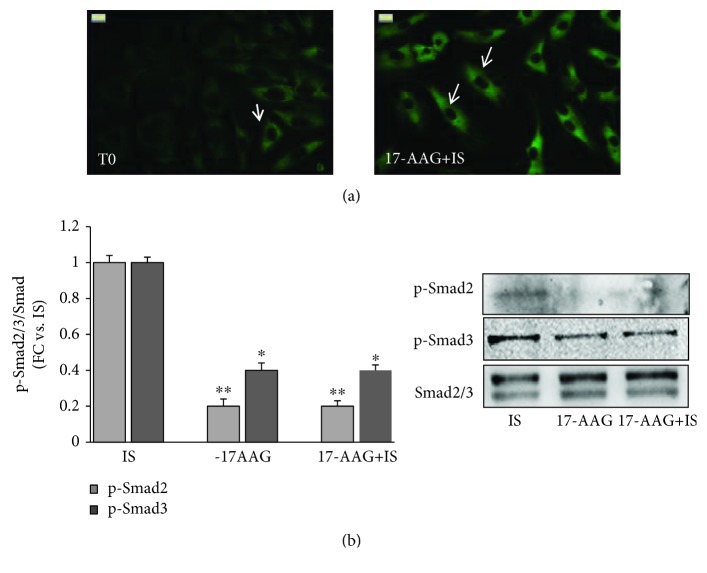
17-AAG blocks HSP90-dependent activation of the Smad pathway induced by IS in NRK-49F cells. (a) HSP90 intracellular localization after a 120-minute IS treatment with or without 17-AAG. Arrows indicate nuclear sites, where no immunopositivity occurred in the presence of 17-AAG. (b) Western blot of Smad 2/3 phosphorylation in cells treated for 120 minutes with IS, 17-AAG, and 17-AAG+IS, respectively. Results are normalized for total Smad 2/3. Values are expressed as fold change ± SEM versus cells treated with IS (^∗^ *p* < 0.05 and ^∗∗^ *p* < 0.01). IS: indoxyl sulfate-treated cells; 17-AAG+IS: cells pretreated with the HSP90 inhibitor 17-AAG and then treated with IS.

We then evaluated the effect of 17-AAG on HSP90 and Smad 2/3 activation. As shown in Figure 6, the pretreatment with 17-AAG blocked, stably over time, the perinuclear and nuclear HSP90 translocation (Figure 6(a)) and the IS-induced phosphorylation of Smad 2/3 (-80% and -60%, *p* < 0.05-0.01) (Figure 6(b)).

## 4. Discussion

The purpose of the present study was essentially to better understand whether moderate IS levels contribute to renal fibroblast activation. First, we set up an in vivo experimental design consisting in a chronic supplementation of moderate amount of IS to mice with a preserved renal function (Supplementary 1); then, to buttress our ex vivo observation, we evaluated in vitro the potential for moderate IS concentration, such as 20 *μ*M, to prime the renal fibroblast phenotype. Finally, since the cytoplasmic receptor of IS, AhR, is tightly regulated by the molecular complex with the chaperone HSP90 [21], we tested whether the profibrotic effects of IS occur through the HSP90/Smad 2/3 pathway.

Our results highlight that even the modest increase in IS, administered continuously for 12 weeks, promotes HSP90 upregulation and *α*-SMA expression, a hallmark of fibroblast transition from a resting phenotype to an activated phenotype, as well as interstitial collagen deposition. When tested on renal fibroblast cell culture, 20 *μ*M IS was able to induce a profibrotic and proinflammatory phenotype, by upregulating the TGF-*β* signal and collagen I synthesis. We also demonstrated that IS-induced fibroblast activation occurs through the HSP90/Smad 2/3-dependent pathway, as proven by the efficacy of the HSP90 inhibitor (17-AAG) in inhibiting IS-induced TGF-*β* signaling and collagen I synthesis.

Among a variety of uremic toxins, IS is of particular interest because its levels are markedly elevated in CKD [6] and it is hardly removed by conventional dialysis due to its protein-binding capacity [22]. Several studies have demonstrated that IS, at uremic concentration (100–500 *μ*M), exerts profibrotic and proinflammatory effects on mesangial [23] and tubular cells [24] and induces epithelial-to-mesenchymal transition in NRK-52E renal proximal tubular cells [25].

Previous animal models on mice and rats were aimed at obtaining the features of a severe renal failure, which include the accumulation of not only IS but also other endogenous compounds [26, 27]; although these models are useful experimental tools to understand the mechanism operating in the uremic condition, they cannot be suggestive when considering a progressive accumulation of IS for small, chronic intake, as occurs in the first stages of the renal disease.

So far, IS has been scarcely investigated in relation to renal fibroblasts and, mostly, when considering moderate IS increases, as those utilized in our experimental setting and found during transition from early to moderate kidney damage [28]. These few studies demonstrate that the exposure to 1-5 mg/lt IS can induce inflammation in renal tubular cells [29] and kidney tissue remodeling through binding and activation of the renal EGF receptor [30]. On this regard, we believe that our animal and in vitro experimental design might offer a good platform to investigate its contribution to renal fibrosis onset in all those conditions that, beside renal failure, may lead to increased level of circulating IS, such as gut microbiota disequilibrium, reduced plasma albumin, and unbalanced IS/albumin ratio for binding competition with other uremic compounds [31].

In the pathogenesis of CKD, resident fibroblasts are key players and renal interstitial fibrosis is considered the hallmark of progressive renal disease. Several studies demonstrate that renal impairment correlates better with interstitial changes than with glomerular changes in most forms of CKD, indicating that renal function is also influenced by the interstitial cell behavior. In 5/6-nephrectomized uremic rats, administration of IS upregulated TGF-*β*, tissue inhibitor of metalloproteinase-1 (TIMP-1), and proalpha 1(I) collagen in the renal cortex and provoked a significant decline in renal function and worsening of renal sclerosis [32].

High IS levels induce ROS production in different cell types, such as vascular endothelial cells, vascular smooth muscle cells, renal tubular cells, monocytes, and macrophages [33–37]. In our setting, the NOX4 mRNA level and ROS production are unchanged by IS treatment, indicating that the IS detrimental effects on our cell line are not induced by oxidative stress damage. This result is reinforced also by ex vivo immunostaining of kidneys for nitrotyrosine, a marker for inflammation and ROS production suitable to detect oxidative damage: increased positivity is detectable in kidneys from IS-treated mice and is mainly localized in tubular cells, while interstitial fibroblasts are negative (Supplementary 3).

A similar observation was obtained from monocytes exposed to different IS concentrations: moderate levels of IS (1-20 *μ*M) evoked only a transient rise in ROS production, but sufficient to promote monocyte differentiation toward a profibrotic and proinflammatory phenotype [5].

The TGF-*β*/Smad 2/3-mediated damage has been found to be operative in several renal fibrotic models [38, 39] and human nephropathies [40]; in the present study, we identify the HSP90/Smad 2/3 activation as the pathway of IS profibrotic induction.

Fibrosis and inflammation constitute a deleterious loop, and the development of fibrosis with loss of renal function often follows renal inflammation [41].

The IS proinflammatory effect was shown in several different cell types: endothelial cells [42], adipocytes [43], and glomerular [23] and renal tubular cells [29]; these studies recognized IS as an important mediator of cell dysfunction in promoting a persistent and systemic inflammatory state in CKD patients. In our experimental setting, we demonstrated that IS can stimulate MCP1 expression in renal fibroblasts and identified HSP90 as a possible shared pawn between IS-induced fibrosis and inflammation in renal fibroblasts.

HSP90 is one of several stress proteins, and as such, its modulation is a potential therapeutic target under stressful conditions. Previous studies demonstrated that modulation of HSP90 affects TGF-*β*-induced collagen synthesis in dermal fibroblasts, [15], attenuates renal fibrosis through degradation of the TGF-*β* type II receptor in TGF-*β*1-treated renal tubular cells and in a murine CKD model [44], regulates the fibroblast activation in pulmonary and hepatic fibrosis [16, 45], and hampers the inflammatory response in atherosclerosis [46] and in ischemia-reperfusion injury in the kidney [47].

To date, the link between HSP90 activation/inhibition and IS effects was scarcely studied. First of all, we have observed that IS induces HSP90 expression; accordingly, pretreatment with 17-AAG blunted Smad 2/3 signaling, inflammatory (MCP1) and fibrotic molecule expression (collagen I, *α*-SMA, and TGF-*β*), and proliferation of renal fibroblasts, suggesting HSP90 activity as a crossroad for IS-induced inflammation and fibrosis.

In conclusion, our report demonstrates that moderate levels of IS cause fibrosis and inflammation by upregulating HSP90 in renal fibroblasts and suggests HSP90 inhibition as an effective tool for reducing IS-induced damage and slowing the progression of renal disease.

## Figures and Tables

**Table 1:** Primer sequences.

Name	Species	Accession number	Forward	Reverse
GAPDH	Rat	NM_017008.4	ctctctgctcctccctgttct	atacggccaaatccgttcaca
Collagen I	Rat	NM_053304.1	tcacctacagcacgcttg	ggtctgtttccagggttg
TGF-*β*	Rat	NM_021578.2	tggaagtggatccacgcgcccaagg	gcaggagcgcacgatcatgttggac
MCP1	Rat	NM_031530.1	cagttaatgccccactcacct	tgacaaatactacagcttctttggg
NOX4	Rat	NM_053524.1	gatgactggaaaccatacaagctaag	catagagcaagtctgcaaaccaactg

## Data Availability

The data used to support the findings of this study are available from the corresponding author upon request.

## Supplementary Materials

Supplementary 1 Figure 1: (A) in vivo experimental design, (B) body weight, and (C) weekly liquid consumption records.

Supplementary 2 Figure 2: the NOX4 mRNA levels are not modified by 1-hour treatment with IS.

Supplementary 3 Figure 3: immunostaining for nitrotyrosine to detect oxidative damage in vivo. Images A-B: magnification 20x; in image B, white boxes indicate the necrotic area. Images C-D: magnification 40x; in image D, black arrows indicate interstitial fibroblasts (negative to the immunostaining).
