# Supplementary figures and images for: Indoxyl Sulfate Induces Renal Fibroblast Activation through a Targetable Heat Shock Protein 90-Dependent Pathway
Source: Oxid Med Cell Longev. 2019 Apr 17;2019:2050183. doi: 10.1155/2019/2050183 (PMC6501427; doi:10.1155/2019/2050183)

## Slide 1
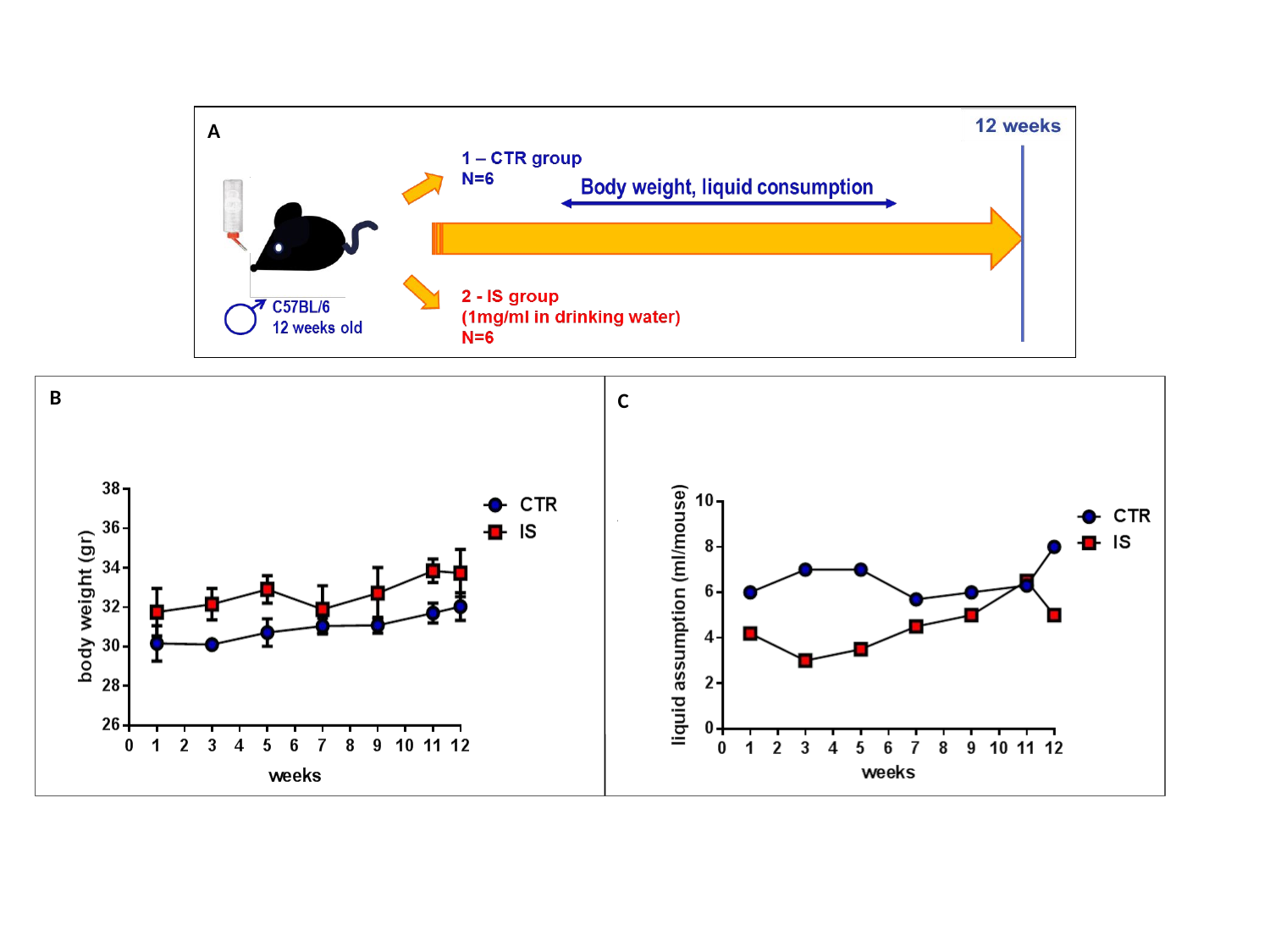

A
B
C

Supplement: Supplementary 1 — Figure 1: (A) in vivo experimental design, (B) body weight, and (C) weekly liquid consumption records. [file 2050183.f1.pptx]

## Slide 1
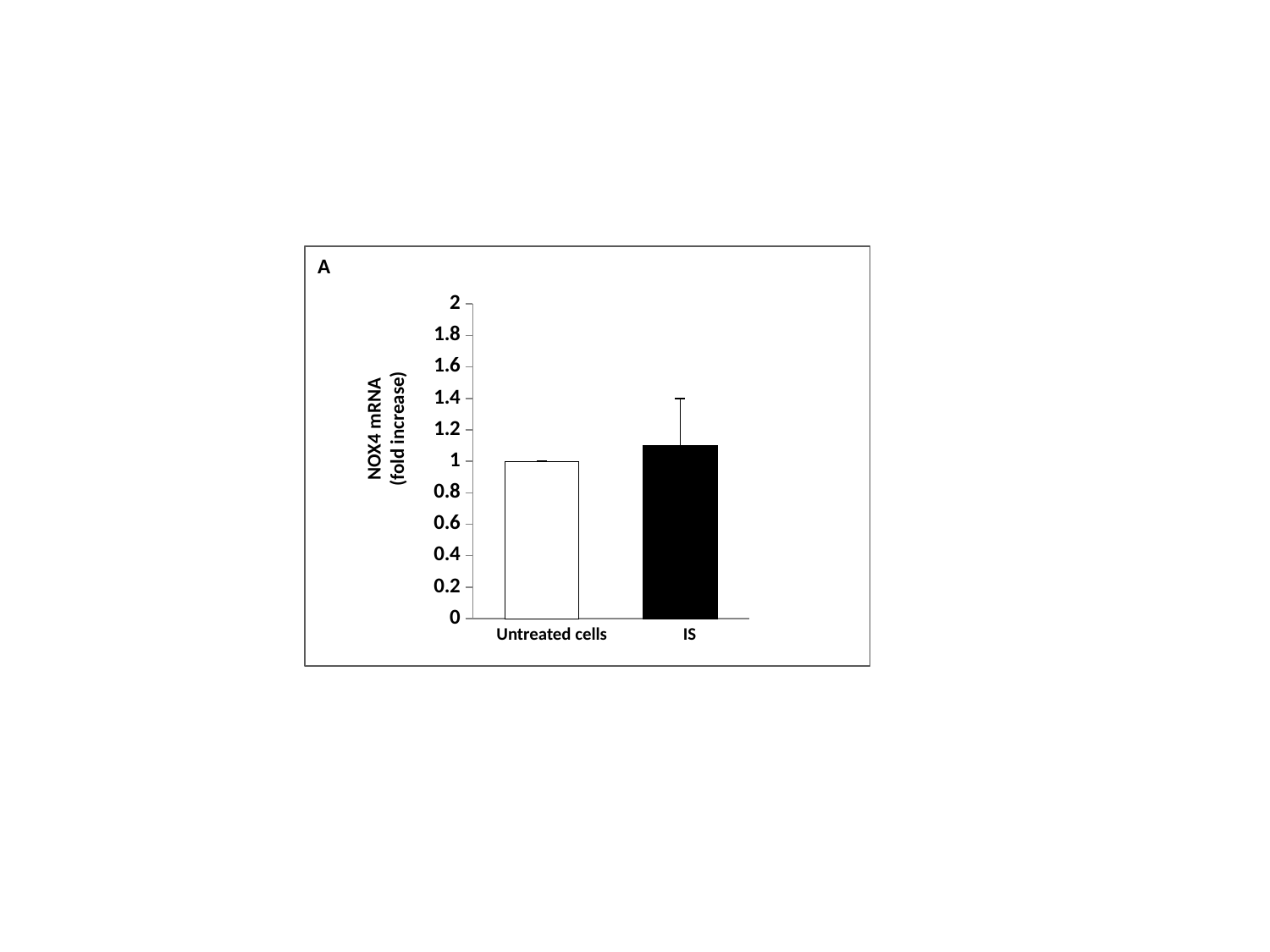

### Chart
| Category | CTR IS AAG IS |
|---|---|
| CTR | 1.0 |
| IS | 1.1 |A
NOX4 mRNA
(fold increase)
 Untreated cells IS

Supplement: Supplementary 2 — Figure 2: the NOX4 mRNA levels are not modified by 1-hour treatment with IS. [file 2050183.f2.pptx]

## Slide 1
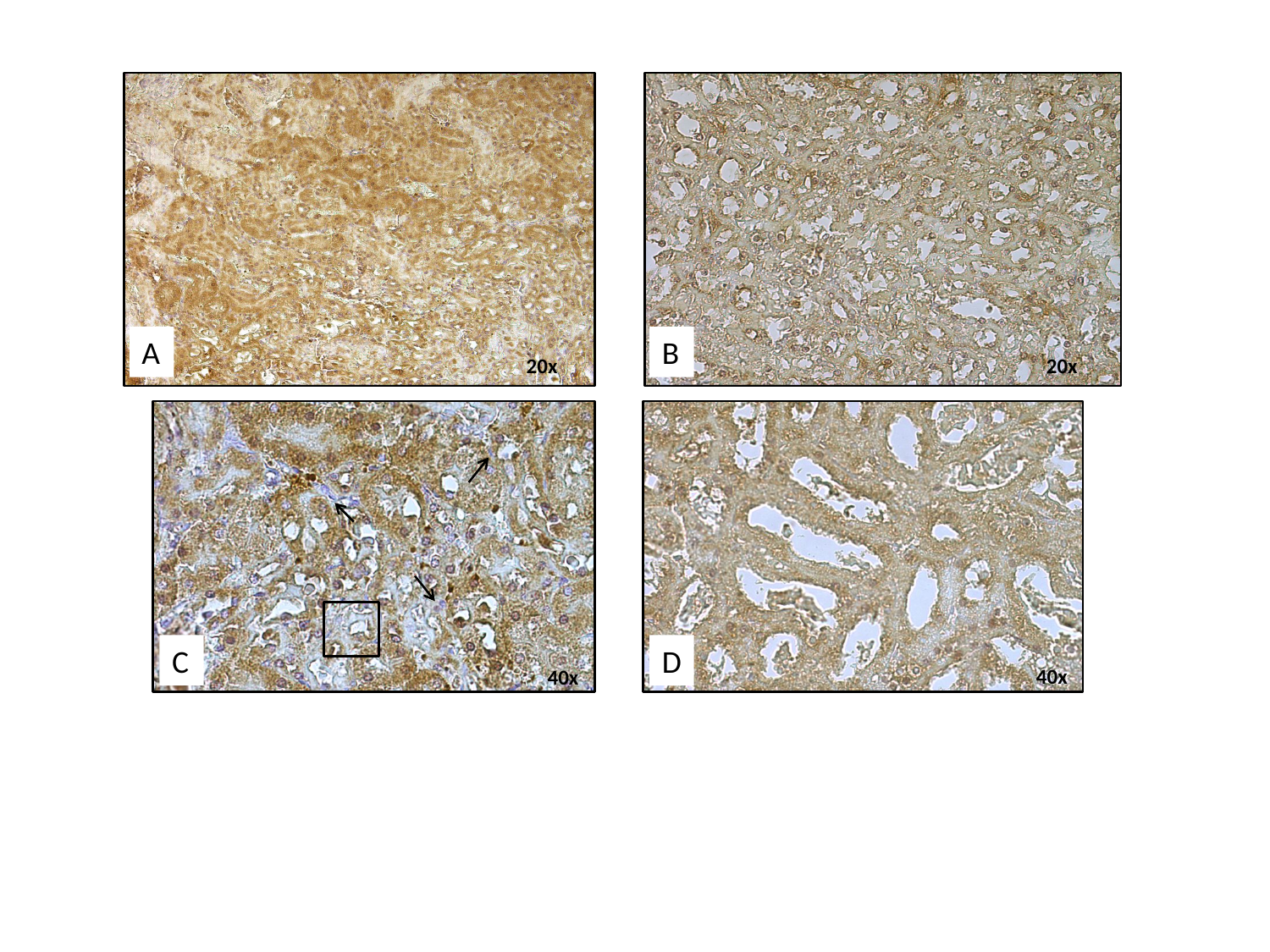

A
B
20x
20x
C
D
40x
40x

Supplement: Supplementary 3 — Figure 3: immunostaining for nitrotyrosine to detect oxidative damage in vivo. Images A-B: magnification 20x; in image B, white boxes indicate the necrotic area. Images C-D: magnification 40x; in image D, black arrows indicate interstitial fibroblasts (negative to the immunostaining). [file 2050183.f3.pptx]
